# The effectiveness of involuntary electroconvulsive therapy (ECT): A population-based study

**DOI:** 10.1192/j.eurpsy.2021.413

**Published:** 2021-08-13

**Authors:** E. Salagre, C. Rohde, K. Ishtiak-Ahmed, C. Gasse, S. Østergaard

**Affiliations:** 1 Bipolar And Depressive Disorders Unit, Hospital Clinic - IDIBAPS - Universitat de Barcelona - CIBERSAM, Barcelona, Spain; 2 Department Of Affective Disorders, Aarhus University Hospital, Aarhus, Denmark; 3 Department Of Clinical Medicine, Aarhus University, Aarhus, Denmark

**Keywords:** Electroconvulsive therapy, Informed Consent, Survival rate, Risk factors, Population Register

## Abstract

**Introduction:**

Involuntary electroconvulsive therapy (ECT) can be a life-saving intervention for patients suffering from potentially lethal conditions who are unable to give informed consent. However, its use is not widespread, probably partly due to the scarce data on hard outcomes following involuntary ECT. In Denmark, involuntary ECT is only used when patients are at imminent/potential risk of dying if not receiving ECT.

**Objectives:**

We aimed to assess the effectiveness of involuntary ECT by estimating the 1-year survival following its administration.

**Methods:**

We conducted a register-based cohort study involving i) all patients receiving involuntary ECT in Denmark between 2008 and 2019, ii) age and sex-matched patients receiving voluntary ECT, and iii) age and sex-matched individuals from the general population. 1-year survival rates were compared via mortality rate ratios.

**Results:**

We identified 618 patients receiving involuntary ECT, 547 patients receiving voluntary ECT, and 3,080 population-based controls. The survival rate in the year after involuntary ECT was 90%. For patients receiving involuntary ECT, the 1-year mortality rate ratios were 3.1 (95% confidence interval (CI)= 1.9-5.2) and 5.8 (95%CI = 4.0-8.2) compared to those receiving voluntarily ECT and to the population-based controls, respectively. Risk factors for early death among patients receiving involuntary ECT were male sex, being ≥70 years old and having organic mental disorder as the treatment indication.

**Conclusions:**

Treatment with involuntary ECT is associated with a high survival rate, suggesting that the intervention is effective. However, patients receiving involuntary ECT constitute a high-risk population that should be monitored closely after this treatment.

**Disclosure:**

No significant relationships.

